# Effects of Collection and Processing Procedures on Plasma Circulating Cell-Free DNA from Cancer Patients

**DOI:** 10.1016/j.jmoldx.2018.07.005

**Published:** 2018-11

**Authors:** Bente Risberg, Dana W.Y. Tsui, Heather Biggs, Andrea Ruiz-Valdepenas Martin de Almagro, Sarah-Jane Dawson, Charlotte Hodgkin, Linda Jones, Christine Parkinson, Anna Piskorz, Francesco Marass, Dineika Chandrananda, Elizabeth Moore, James Morris, Vincent Plagnol, Nitzan Rosenfeld, Carlos Caldas, James D. Brenton, Davina Gale

**Affiliations:** ∗ Cancer Research UK Cambridge Institute, Li Ka Shing Centre, Robinson Way, University of Cambridge, Cambridge, United Kingdom; † Department of Cancer Genetics, Institute for Cancer Research, Oslo University Hospital, Norway; ‡ Department of Pathology, Oslo University Hospital, Oslo, Norway; § Cancer Research UK Major Centre, Cambridge, United Kingdom; ¶ Cambridge Breast Unit, Cambridge University Hospitals NHS Foundation Trust, Addenbrooke's Hospital, Cambridge, United Kingdom; ‖ Department of Oncology, Cambridge University Hospitals NHS Foundation Trust, Addenbrooke's Hospital, Cambridge, United Kingdom; ∗∗ UCL Genetics Institute, University College London, London, United Kingdom

## Abstract

Circulating tumor DNA (ctDNA) offers new opportunities for noninvasive cancer management. Detecting ctDNA in plasma is challenging because it constitutes only a minor fraction of the total cell-free DNA (cfDNA). Pre-analytical factors affect cfDNA levels contributed from leukocyte lysis, hence the ability to detect low-frequency mutant alleles. This study investigates the effects of the delay in processing, storage temperatures, different blood collection tubes, centrifugation protocols, and sample shipment on cfDNA levels. Peripheral blood (*n* = 231) from cancer patients (*n* = 62) were collected into K_3_EDTA or Cell-free DNA BCT tubes and analyzed by digital PCR, targeted amplicon, or shallow whole-genome sequencing. To assess pre-analytic effects, plasma was processed under different conditions after 0, 6, 24, 48, 96 hours, and 1 week at room temperature or 4°C, or using different centrifugation protocols. Digital PCR showed that cfDNA levels increased gradually with time in K_3_EDTA tubes, but were stable in BCT tubes. K_3_EDTA samples stored at 4°C showed less variation than room temperature storage, but levels were elevated compared with BCT. A second centrifugation at 3000 × *g* gave similar cfDNA yields compared with higher-speed centrifugation. Next-generation sequencing showed negligible differences in background error or copy number changes between K_3_EDTA and BCT, or following shipment in BCT. This study provides insights into the effects of sample processing on ctDNA analysis.

Circulating tumor DNA (ctDNA) in plasma offers new opportunities for noninvasive cancer management. Recent studies have demonstrated its potential for molecular stratification, monitoring tumor response, identifying resistance mutations, and patients at risk of relapse.^1^, ^2^ Detecting ctDNA in plasma is challenging because it constitutes only a minor fraction of the total cell-free DNA (cfDNA), particularly in early-stage cancers and in the minimal residual disease setting.^3^, ^4^ A proportion of background wild-type DNA is believed to originate from lysis of white blood cells.^5^ Previous studies have highlighted the pre-analytic effects of different processing and collection protocols on plasma ctDNA levels from cancer patients and pregnant women.^6^, ^7^, ^8^, ^9^ On the basis of these results, it is recommended to process whole-blood samples for retrieval of plasma as soon as possible after collection, before *in vitro* cell lysis. At the same time, a double-centrifugation protocol has been recommended to obtain cell-free plasma, using an initial slow centrifugation speed to separate plasma, then fast centrifugation to clear cellular material.^7^ However, some of these procedures may be difficult to perform in a clinical setting due to lack of appropriate personnel or equipment. To circumvent this, cell-stabilizing blood collection tubes have become available to stabilize cfDNA, enabling a delay in processing, which may be done under more controlled conditions and within centralized laboratories. This study performed a systematic comparison of the effects of different processing protocols and collection tubes on the levels of cfDNA and ctDNA from cancer patients using digital PCR (dPCR). With the growing use of next-generation sequencing (NGS) for the analysis of ctDNA, the effect of different protocols and collection tubes on the performance of targeted amplicon and shallow whole-genome sequencing (sWGS) for quantification of plasma DNA was also investigated.

## Materials and Methods

### Analysis Modules

The study was designed to include five different modules: Module 1 investigated the effects of delayed processing on the levels of circulating DNA (cfDNA and ctDNA) in plasma collected in K_3_EDTA tubes (9 mL S-Monovette; Sarstedt, Nümbrecht, Germany). The separation of plasma was delayed for different durations: 0, 6, 24, 48, and 96 hours, and 1 week at room temperature (19°C to 25°C). Module 2 investigated the effects of storage temperature on the levels of circulating DNA in plasma collected in K_3_EDTA tubes. Samples were stored at room temperature or at 4°C before processing at the following hours post-collection: 0, 24, 48, and 96 hours. Module 3 investigated the effects of collection devices on the levels of circulating DNA. Blood samples from each patient were collected at the same time point into K_3_EDTA tubes and cell-stabilization blood collection tubes (10 mL Cell-Free DNA BCT; Streck, La Vista, NE), respectively. BCTs contain a proprietary formaldehyde-free preservative that stabilizes nucleated blood cells preventing the release of genomic DNA.^10^, ^11^ The samples were processed at the following times post-collection: 0, 96 hours, and 1 week. Module 4 investigated the effects of different centrifugation protocols on the levels of circulating DNA. Module 5 investigated the effects of shipment on samples collected in BCT tubes at ambient temperature. For modules 1, 2, 3, and 5, plasma was separated from blood using a double-centrifugation protocol (protocol A): a first centrifugation at 820 × *g* for 10 minutes in a mega-centrifuge (Thermo Sorvall Legend RT; Thermo Fisher Scientific, Waltham, MA), then subjected to a second centrifugation step of the plasma supernatant at 14,000 × *g* for 10 minutes in a benchtop micro-centrifuge (Heraeus Fresco 21; Thermo Fisher Scientific). For module 4, blood aliquots from the same patients were processed with three different protocols: protocol A as above, protocol B with the first centrifugation performed at 1600 × *g* and the second centrifugation at 14,000 × *g* for 10 minutes in a bench top micro-centrifuge, and protocol C with both first and second centrifugations performed in the same mega-centrifuge, initially at 1600 × *g* for 10 minutes, then at 3000 × *g* for 10 minutes.

### Patient Samples and DNA Extractions

Peripheral whole blood was collected from 62 patients in total: 47 patients with high-grade serous ovarian cancer and 15 patients with metastatic breast cancer. Informed consent was obtained from each patient with protocols approved by an institutional ethics committee. Fifteen to 30 mL blood from each patient was processed according to each analysis module. DNA from all samples, except module 5, was extracted from an average 1.4 mL (range, 0.3 to 2.76 mL) plasma using the QIAamp Circulating Nucleic Acid Kit (Qiagen, Hilden, Germany) according to the manufacturer's protocol, except that 6.2 μg of carrier RNA was added per sample. DNA was eluted twice through the column to maximize yield. A nonhuman spike-in PCR product was added to each sample as an internal quality control to assess extraction efficiency.^12^ In module 5, DNA was extracted from plasma on a QIAsymphony robot (Qiagen) using a 2-mL extraction protocol. Eluted DNA was stored at −20°C until analysis.

A total of 231 blood samples aliquots were analyzed in this study. Table 1 summarizes the number of plasma samples collected for each module. Note that the collection was designed in such a way that each sample from every processing condition (temperature, collection tube, delayed processing duration) had a matched sample that was collected in K_3_EDTA and processed immediately (denoted E.RT.0h) using centrifugation protocol A, and was assigned as the reference sample for each condition. The levels of circulating DNA (either cfDNA or ctDNA), were expressed as a ratio of the respective data with the reference sample (E.RT.0h). Therefore, data collected under the same processing conditions could be grouped together to evaluate the effect of the processing even though they were collected from different patients. A more detailed summary of the distribution of samples involved in each module is given in Supplemental Table S1.

**Table 1:** Summary of Data Available for Different Modules

Module	Collection devices	Temperature	Delay before sample processing					
			0 hours	6 hours	24 hours	48 hours	96 hours	1 week
Module 1	EDTA	Room temperature	26	21	20	10	5	5
Module 2	EDTA	Room temperature/4°C	26	21	20/11	10/10	5/5	5
Module 3	EDTA/BCT	Room temperature	10/5	-	-	-	5/10	5/15
Module 4	EDTA	Room temperature	13	-	-	-	-	-
Module 5	EDTA/BCT	Room temperature	13/-	-	-	-/10	-/2	-/1∗

Module 1: The effects of delayed processing.

Module 2: The effects of storage temperature. Samples were stored both in room temperature and at 4°C. 20/11 indicates that 20 tubes were stored at room temperature and 11 at 4°C, and so on.

Module 3: The effects of collection devices (EDTA versus BCT).

Module 4: The effects of different centrifugation speeds.

Module 5: The effects of shipment in BCT.

Dashes indicate no data available.

### Quantification of Circulating Plasma DNA by dPCR and Targeted Amplicon Sequencing

Plasma samples from ovarian and breast cancer patients were first quantified by dPCR (using the Biomark microfluidic system (Fluidigm, South San Francisco, CA) as previously described,^13^ using an assay that targets a 65-bp amplicon in *RPP30*, a nonamplified region in the genome, to estimate cfDNA levels.^12^, ^14^ ctDNA levels were then determined by dPCR using dual-labelled patient-specific TaqMan assays designed to mutant and wild-type sequences in *TP53* or *PIK3CA*, or deletions in chromosome 8, 11, or 17. A summary of the samples analyzed is provided in Supplemental Table S1, and sequences of primers and fluorescent probes, amplicon sizes, and amplification conditions used in dPCR are detailed in Supplemental Table S2.

The levels of cfDNA and ctDNA were calculated from the number of observed amplifications above a set threshold, and Poisson statistics were used to convert the number of observed amplifications to estimated targets, assuming independent segregation of DNA molecules into the microfluidic reaction chambers. The total number of amplifiable copies of DNA molecules per mL of plasma (copies/mL) were calculated, taking into account the relative fraction of the extracted DNA loaded and the proportion of sample lost during the loading process through the microfluidic channels. The levels of ctDNA were calculated as mutant allele fraction (ie, the fraction of mutant DNA copies divided by the total cfDNA copies) expressed as a percentage or as mutant copies/mL plasma. For the purpose of comparing different protocols in the modules, the data are expressed at each processing condition as a ratio from the E.RT.0h reference sample that was collected in K_3_EDTA and immediately processed according to protocol A, unless otherwise specified.

To investigate the effects of different collection devices and processing protocols on the performance of NGS, plasma samples from all modules were analyzed by Tagged Amplicon deep sequencing (TAm-Seq), as previously described.^13^ TAm-Seq is a targeted amplicon sequencing method that allows identification and quantification of low-frequency mutant alleles in plasma across sizable genomic regions. Sequencing was performed using an Illumina HiSeq 2500 sequencer (Illumina, San Diego, CA) to an average of greater than 1000× sequencing depth. Mutations were identified and quantified as previously described.^13^ To assess the effect of collection and processing procedures on the background error rates during NGS, the allelic read ratio (reference/alternative) was generated at each position within R software version 3.1.2^15^ from the BAM files, using the Bioconductor 3.2 software packages Rsamtools version 1.22.0 and Biostrings version 2.38.4.^16^ All positions flagged as polymorphic by the 1000 Genomes Project (*http://www.internationalgenome.org*, last accessed) or the COSMIC database (*https://cancer.sanger.ac.uk/cosmic*, last accessed), were filtered out.

To investigate the effects of shipping on global somatic copy number alterations, samples in module 5 were also subjected to sWGS.^17^ Briefly, a DNA library was prepared from 2 to 10 ng of cfDNA from each sample using the ThruPLEX DNA-seq Kit (Takara Bio, Inc., Shiga, Japan) and sequenced on an Illumina HiSeq 4000 to 0.1× average depth using single-end sequencing. Sequence data were analyzed using a pipeline that involved the following: single-end sequence reads were aligned to the human reference genome (GRCh37) using BWA-mem software version 0.7.17^18^ after removing any contaminant adapter sequences. SAMtools software version 1.7 (*http://samtools.sourceforge.net*)^19^, ^20^ was used to convert files to BAM format. PCR and optical duplicates were marked using Picard-Tools' MarkDuplicates software feature version 2.17.6 (*https://broadinstitute.github.io/picard*), and these were excluded from downstream analysis along with reads of low mapping quality and supplementary alignments. Reads in each sample were down-sampled to approximately 3 million reads to have similar coverage between patients and conditions. Subsequently, copy number analysis was performed in R^15^ using the R package CNAclinic version 1.0 (*https://github.com/sdchandra/CNAclinic*, last accessed December 21, 2017), a software suite that allows for robust copy number analysis of sWGS data. Briefly, sequence reads were allocated into equally sized (100 Mb) nonoverlapping bins throughout the length of the genome. Read counts in each bin were corrected to account for sequence GC content and mappability, and regions corresponding to artifacts and probable germline changes were excluded from downstream analysis utilizing a cohort of 45 healthy controls. After median normalization, binned counts were segmented using both the Circular Binary Segmentation– and Hidden-Markov Model–based algorithms, and an averaged log_2_ R value per bin was calculated.

### Statistical Analysis

The difference in circulating DNA levels between different subgroups in each module was analyzed using nonparametric Mann–Whitney rank sum test unless specified, and *P* < 0.05 was considered statistically significant. To assess the noise of sWGS data, values corresponding to the median of the absolute values of all pairwise differences were calculated between log_2_ R copy numbers. This metric provides a measure of the noise of the sample that is less dependent on true biological copy number variation and more on technical variation.^21^ To compare the three collection methods in all patients, pairwise Spearman correlations were calculated between the binned copy number segments of the three collection methods. Furthermore, a nonparametric Wilcoxon signed rank test was applied on these values to test the similarity of the copy number profiles between all pairwise samples.

## Results

### Module 1: The Effects of Delayed Processing on the Levels of Circulating DNA in Plasma Collected in EDTA Tubes

**Figure 1:**
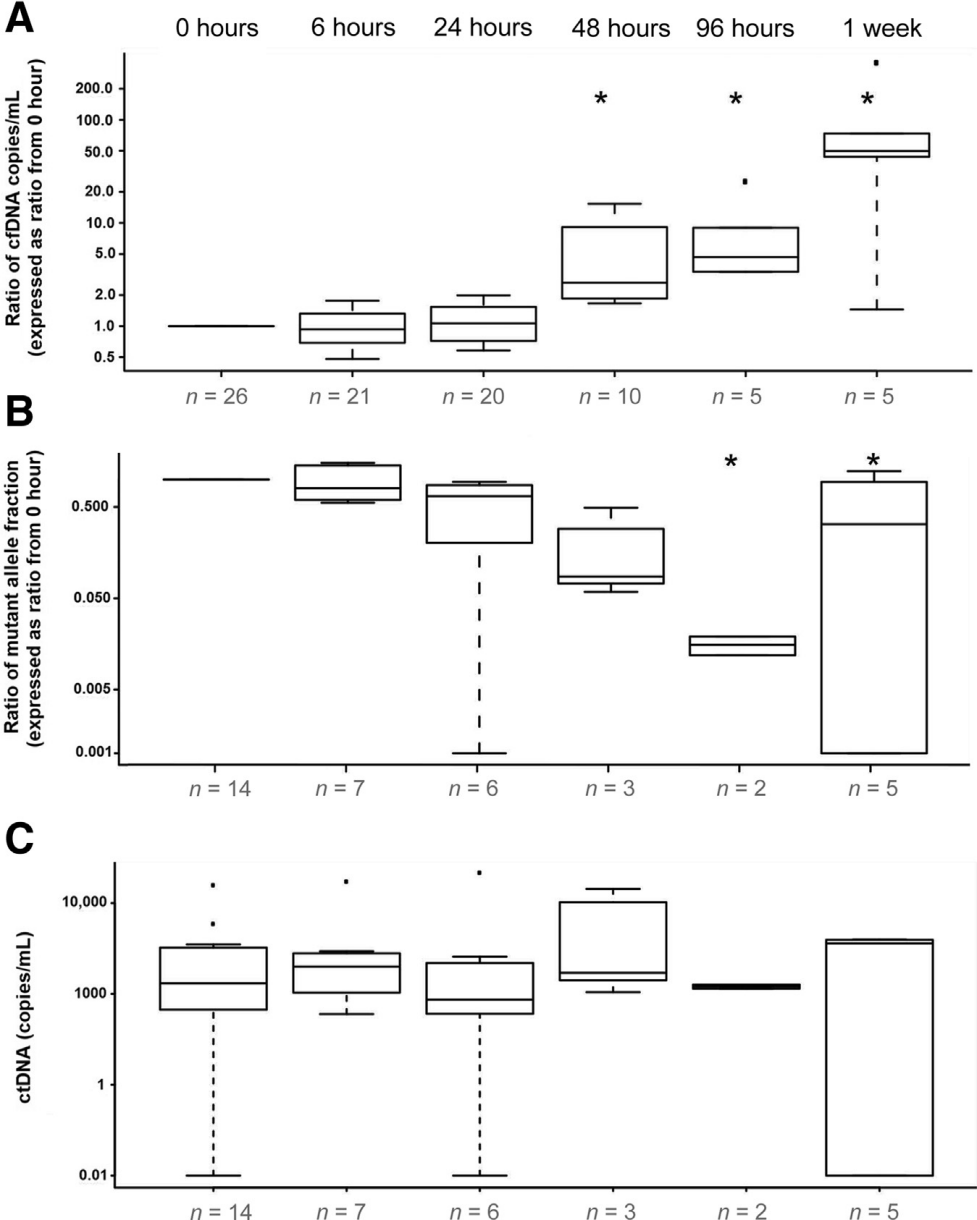
The effects of delayed processing on the levels of circulating DNA in plasma collected in K_3_EDTA tubes. Blood samples were collected into K_3_EDTA tubes and stored at room temperature for 0, 6, 24, 48, and 96 hours, and 1 week before plasma separation. Cell-free DNA (cfDNA) copies/mL plasma (**A**), mutant allele fraction (**B**). **C:** Circulating tumor DNA (ctDNA) copies/mL plasma in samples processed at different time of delay. The bottom and top of the box represent the first and third quartiles, respectively, and the band inside the box represents the median. Data are expressed as the ratio from E.RT.0h of each patient's immediately processed K_3_EDTA sample. ^∗^*P* < 0.05 versus E.RT.0h (Mann–Whitney rank sum test).

In this module, all samples (*n* = 26) were collected in K_3_EDTA tubes. One tube from each collection was processed immediately. The other tubes were stored at room temperature and processed at different prolonged time points: 6, 24, 48, 96 hours, and 1 week. Analysis by dPCR showed that the levels of cfDNA in the plasma samples increased gradually with increasing delay in the processing (Figure 1A), whereas the fraction of ctDNA decreased (Figure 1B). In particular, the levels of cfDNA increased significantly after 48, 96 hours, and 1 week of delay, whereas the mutant allele fraction of ctDNA decreased significantly after 96 hours and 1 week of delay (Mann–Whitney rank sum test, *P* < 0.05). Previous reports have indicated that in analysis of circulating cell-free DNA from maternal plasma, despite changes in total cfDNA, the levels of fetal DNA are relatively stable in different storage and processing conditions.^8^, ^22^ Indeed, our results confirm that the numbers of mutant molecules, expressed as copies/mL of plasma, were relatively stable across the different processing time points with no statistically significant difference observed compared to samples that were processed immediately (Figure 1C and Supplemental Figure S1).

### Module 2: The Effects of Storage Temperature on the Levels of Circulating DNA in Plasma Collected in K_3_EDTA Tubes

**Figure 2:**
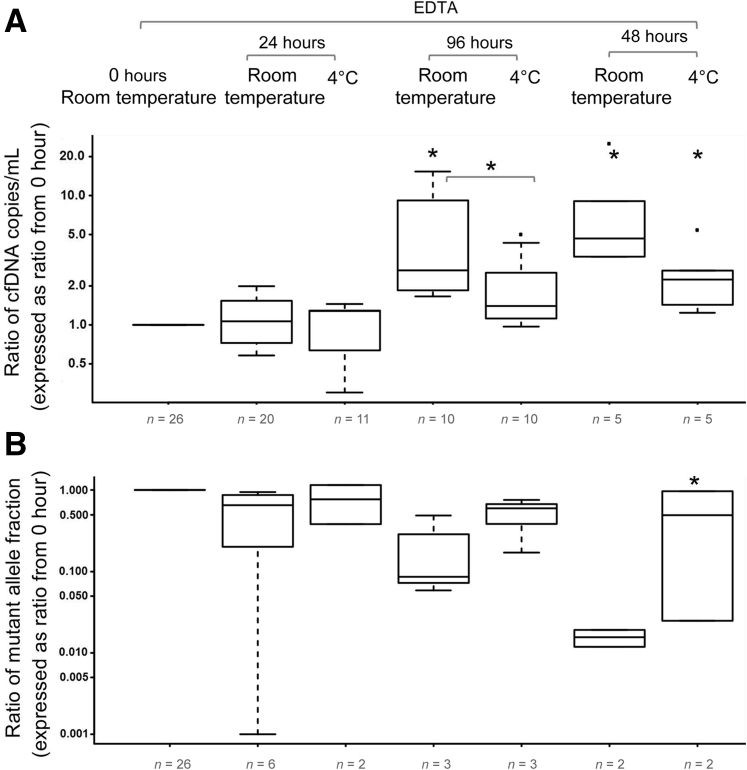
The effects of storage temperature on the levels of circulating DNA in plasma collected in K_3_EDTA tubes. Blood samples collected into K_3_EDTA tubes were stored at room temperature and at 4°C for 24, 48, and 96 hours, and 1 week before plasma was separated. Cell-free DNA (cfDNA) copies/mL plasma (**A**) and mutant allele (**B**) fraction. The bottom and top of the box represent the first and third quartiles, respectively, and the band inside the box represents the median. Data are expressed as the ratio from E.RT.0h of each patient's immediately processed K_3_EDTA sample. ^∗^*P* < 0.05 versus E.RT.0h (Mann–Whitney rank sum test).

In this module, all samples (*n* = 26) were collected in K_3_EDTA tubes and either processed to plasma immediately or after 24, 48, and 96 hours. The individual tubes were stored in two conditions: at room temperature (19°C to 25°C) or at 4°C. If kept at room temperature, dPCR showed that the levels of cfDNA significantly increased after 48 hours. If kept at 4°C, the levels increased after 48 hours but were significantly lower than those observed at room temperature (Figure 2A). If delayed for 96 hours, samples kept at room temperature and 4°C all increased significantly. The changes in mutant allele fraction showed an inverted similar trend, although the amount of available data were too low for statistical analysis (Figure 2B).

### Module 3: The Effects of Collection Devices (K_3_EDTA versus Cell-Free DNA BCT) on the Levels of Circulating DNA

**Figure 3:**
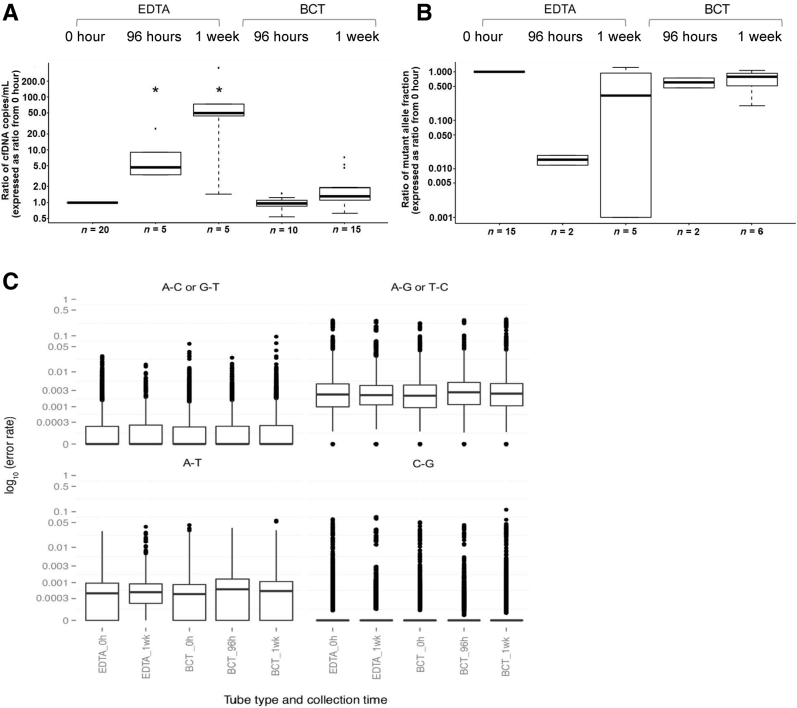
The effects of collection device (K_3_EDTA versus BCT) on the levels of circulating DNA. Blood samples collected into K_3_EDTA tubes were processed immediately, after 96 hours or 1 week at room temperature. Blood samples in BCT were stored at room temperature for 96 hours and 1 week before plasma separation. Cell-free DNA (cfDNA) copies/mL plasma (**A**) and mutant allele (**B**) fraction. **C:** The distributions of the ratio of nonreference/reference alleles as generated by targeted amplicon sequencing shown in boxplots. The bottom and top of the box represent the first and third quartiles, respectively, and the band inside the box represents the median. Data are expressed as the ratio from E.RT.0h of each patient's immediately processed K_3_EDTA sample (**A** and **B**) or log_10_ scale (**C**). ^∗^*P* < 0.05 versus E.RT.0h (Mann–Whitney rank sum test).

In this module, one K_3_EDTA tube for each collection was processed immediately (E.RT.0h) and served as a reference sample (*n* = 20). The other K_3_EDTA tubes were stored for 96 hours (*n* = 5) and 1 week (*n* = 5) at room temperature. Cell-free DNA BCT's were stored at room temperature and processed immediately (*n* = 5) or delayed for 96 hours (*n* = 10) and 1 week (*n* = 15). The cfDNA levels increased significantly after 1 week if kept in K_3_EDTA tubes, but remained at similar levels if kept in BCT (Figure 3A). The changes in the mutant allele fraction showed an inverted similar trend, but the amount of data available were too low for statistical analysis (Figure 3B). The mutant allele fraction from six patients that were collected in K_3_EDTA and processed immediately, versus the matched samples that were collected in BCT was compared and processed after 1 week's delay. The levels of ctDNA were similar for four patients but decreased twofold for two patient samples (Supplemental Figure S2). There was no statistically significant difference in the numbers of mutant copies/mL plasma between storage in the two tube types (Supplemental Figure S1).

The effects of collection and processing procedures on the background error rates during NGS analysis were next assessed using targeted amplicon sequencing. As previously described, different A/C/G/T base substitutions are associated with different error rates.^13^ The distribution of the ratio of nonreference/reference alleles was plotted as box plots, shown according to mutation types. No difference was observed using different collection devices and processing conditions (Figure 3C).

### Module 4: The Effects of Different Centrifugation Speeds on the Levels of Circulating DNA

**Figure 4:**
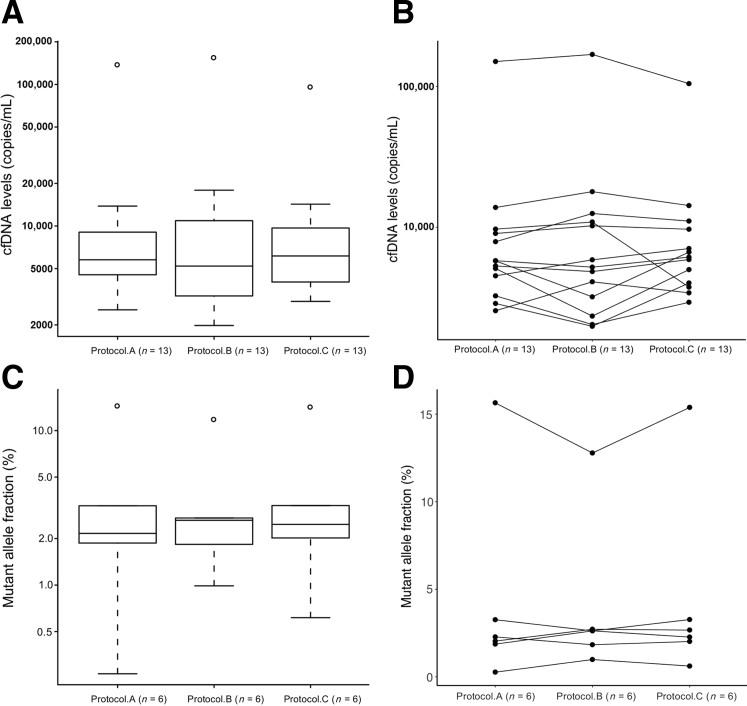
The effects of different centrifugation speeds on the levels of circulating DNA. Blood samples were collected into K_3_EDTA tubes and processed to plasma with three different protocols. All protocols included two 10-minute centrifugation steps, the first on whole blood, and the second on plasma aliquots. Protocol A (820 and 14,000 × *g*), protocol B (1600 and 14,000 × *g*), Protocol C (1600 and 3000 × *g*). Cell-free DNA (cfDNA) copies/mL plasma (**A** and **B**) and mutant allele (**C** and **D**) fractions (%) in samples processed by different protocols. The bottom and top of the box represent the first and third quartiles, respectively, and the band inside the box represents the median.

In this module, all samples (*n* = 13) were collected in K_3_EDTA tubes and processed immediately. Aliquots from the same patients were processed using three different centrifugation protocols (A to C) as defined in Materials and Methods. There were no statistically significant differences across the three protocols on the total circulating DNA levels as measured by dPCR (Figure 4, A and B), or in mutant allele fraction as measured by targeted amplicon sequencing (Figure 4, C and D).

### Module 5: The Effects of Shipment of cfDNA BCT on Mutant Allele Fraction and Global Copy Number Changes

**Figure 5:**
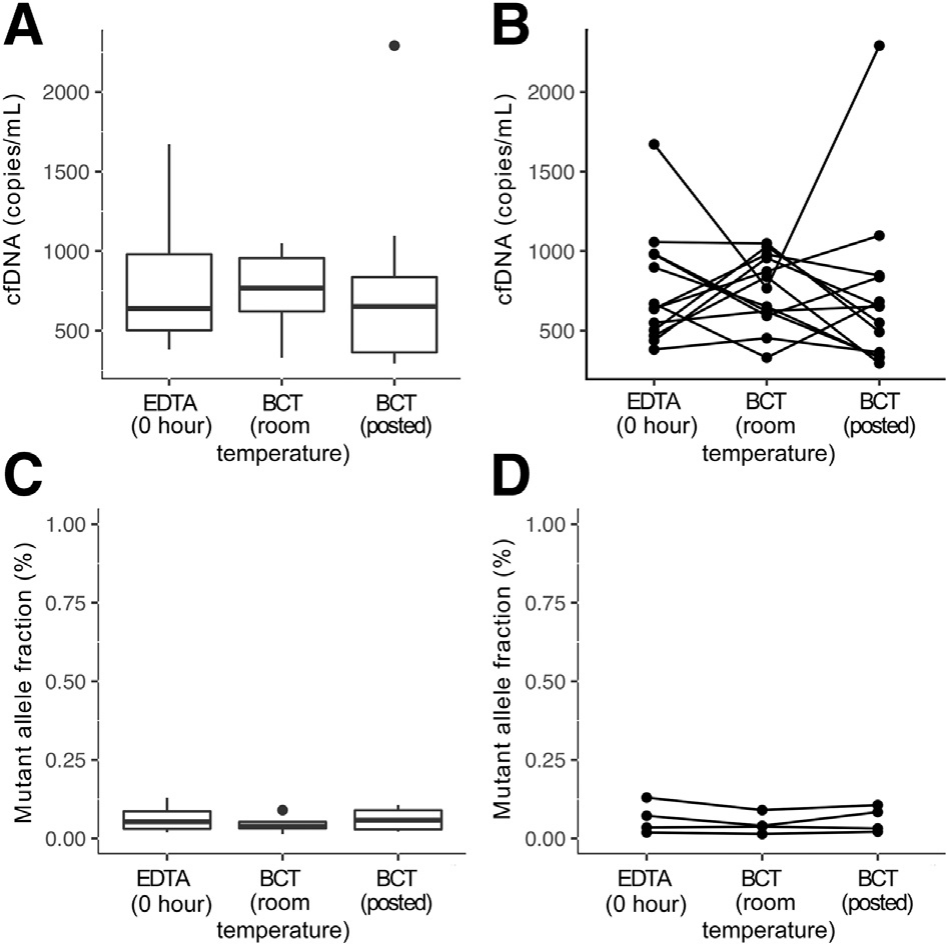
The effects of shipping using cell-free DNA BCT on the levels of circulating DNA. Blood samples were collected in K_3_EDTA and cell-free DNA BCT tubes, and processed immediately except for one cell-free DNA BCT from each collection that was shipped by mail back to the same collection center [BCT (posted)]. Cell-free DNA (cfDNA) levels (copies/mL) (**A** and **B**) and mutant allele (**C** and **D**) fractions. The bottom and top of the box represent the first and third quartiles, respectively, and the band inside represents the median.

In this module, three tubes of blood were drawn from each patient (*n* = 13). K_3_EDTA tubes were processed immediately (E.RT.0h), one cell-free DNA BCT was collected and stored at room temperature within the same centralized processing laboratory, whereas the other BCT was packaged and shipped back to the same laboratory. All shipped samples, apart from three, were received and processed within 48 hours from the time of collection. Of these, two BCTs were processed after 96 hours and one was processed after 5 days. The stored BCTs were processed at the same time as the matched shipped sample. There was no statistically significant difference in cfDNA levels between the three collection methods (Figure 5, A and B). *TP53* mutations were identified by TAm-Seq in four patients, and there were no statistically significant differences in mutant allele fraction using the different collection methods (Figure 5, C and D).

To further investigate the effects of collection methods on global copy number changes, sWGS analysis was performed on four patients with detectable *TP53* mutations (P161, P227, P479, P488) and four without (P615, P489, P464, P450). Data from one patient (P464) were excluded from further analysis because the total read count generated for one of the collection methods was below 1 million. This is below the threshold recommended for inference when analyzing shallow coverage.^23^ The segmental copy number profiles among the three collection methods were highly similar, showing an average Spearman correlation of 0.76, range 0.44 to 0.98 (Supplemental Figure S3 and Supplemental Table S3). The paired Wilcoxon test *P* values indicated no significant differences in all 21 copy number distributions comparisons (*P* > 0.001). Supplemental Figure S4 shows an example of the copy number alterations in plasma samples processed with and without shipping. The same gains and losses in chromosomal arms were identified in all three protocols. Supplemental Figure S5 depicts the estimation of noise in the sWGS data using values that were the median of the absolute values of all pairwise differences. All patients showed very similar noise levels between the different tubes and protocols.

## Discussion

Multiple research studies have demonstrated the potential of using plasma as a tool for noninvasive cancer management. There is increasing interest in incorporating ctDNA as a liquid biopsy in both clinical and research settings. Because the frequency of mutant alleles in plasma may be low, particularly in early-stage disease, it is crucial to optimize and standardize pre-analytic sample processing procedures to maintain the quality of samples for accurate quantification of rare mutant molecules. In this study, the pre-analytic effects of blood sample processing procedures, including the use of different blood collection tubes, storage conditions, and centrifugation speeds, were examined on downstream analysis of cfDNA using different molecular technologies including dPCR, targeted amplicon, and genome-wide sequencing. Our results show that levels of cfDNA are stable in K_3_EDTA tubes at room temperature for up to 24 hours. If delayed beyond 24 hours, storage of K_3_EDTA blood at 4°C appeared to delay the increase in background cfDNA. It is worth noting that a recent study demonstrated that storing the samples in K_2_EDTA tubes at 4°C kept the cfDNA levels stable for a course of 3 days.^24^ This agrees with the observations that storing K_3_EDTA tubes at 4°C improved the stability of cfDNA compared with room temperature storage. Alternatively, collection into cell-free DNA BCT tubes at room temperature maintained stable cfDNA levels for at least a week. These tubes can facilitate delayed and centralized blood processing, circumventing issues arising with delayed plasma processing. Other researchers have evaluated alternative cell-stabilization tubes such as CellSave (CellSearch system; Menarini Silicon Biosystems, Huntington Valley, PA) and PAXgene Blood ccfDNA tubes (Qiagen) and demonstrated similar stability when sample processing was delayed.^9^, ^25^ New cell-free stabilization tubes have recently become available [eg, Cell-free DNA Collection tube (Roche, Basel, Switzerland), cf-DNA Preservation tube (Norgen Biotek, Thorold, ON, Canada), Blood STASIS 21-ccfDNA, (MagBio Genomics, Gaithersburg, MD), and LBgard Blood tubes, Biomatrica, San Diego, CA)], and it will be important to test these thoroughly to assess their performance for optimal sample processing procedures before next-generation sequencing and dPCR analysis of ctDNA.

These findings have addressed a few of the practical challenges in the blood-to-plasma sample processing workflow in a hospital setting. For example, in the clinic, processing may be delayed due to shortage of staff to enable immediate processing, or collection outside office hours. In some scenarios, when conducting multicenter clinical trials, many individual centers do not have access to the full spectrum of centrifuges with the higher second centrifugation speeds required to perform the recommended double-centrifugation procedures. The ability to delay processing by collecting into cell-stabilization tubes, or the flexibility to perform the centrifugation in a range of different types of centrifuges, or storing at 4°C after collection for a short period, will greatly improve the feasibility of collecting high-quality specimens. For samples collected across a wide geographical area, shipment may be necessary before central processing to standardize pre-analytic factors and maximize cost-effectiveness. This study showed no statistically significant difference in NGS background noise with or without shipment. However, other studies have shown that the shipping temperature of cell-free DNA BCT was deemed to be a critical factor to ensure delivery of high-quality specimens for downstream ctDNA analysis.^26^ In these studies, variable results were observed at extreme temperatures, at ≤10°C and 40°C, which affected the cellular interface, resulted in an elevated ratio of long/short genomic DNA fragments, and a decrease in plasma volume. These studies indicate that shipment temperature should be carefully controlled by the use of insulated packages, gel blocks, or temperature logging devices to maintain stability.

Previous studies have mainly focused on locus-specific analysis using quantitative PCR or dPCR that examined one locus at a time. With technology advances, an increasing number of molecular profiling strategies have been developed using NGS,^27^ which provides a higher resolution and larger genomic coverage than a locus-specific approach. It is therefore important to also understand the effects of cfDNA sample processing on the analytical performance of NGS-based analysis. It is particularly important to test whether using a collection tube containing a preservative has the potential to introduce DNA sequence modifications, which may be misinterpreted as true patient-specific genomic alterations. A recent study examined the influence of sample collection in CellSave tubes on the analysis of global copy number variations using NGS technology, and did not find differences in allele frequencies compared with EDTA blood.^9^ In this study with BCT and K_3_EDTA tubes, the effects of processing on the background error rates during targeted amplicon sequencing and sWGS were evaluated. As expected, different error rates were observed in different base substitutions, but there was no difference in background error rate regardless of the type of collection device and sample processing schedule. The sWGS analysis results agreed with previous findings in that copy number data were consistent across conditions.^28^

All of these findings provide important insights for the potential incorporation of routine NGS technology in plasma-based molecular diagnostics. Beyond the analysis of ctDNA, it is crucial to also understand the impact of pre-analytical factors on other nucleic acids or genomic variants, such as tumor-specific RNA (ctRNA), microRNA, or DNA methylation, some of which have been studied,^29^ but more evidence is required. Their quantification would likely be affected by the levels of total RNA or methylated DNA that is derived from the blood cells. It is important to understand whether the effects of sample processing procedures could be addressed in a similar manner to the effects on circulating DNA.

With the increasing understanding of genomic alterations and matched targeted treatment options, the demand for a non-invasive molecular profiling tool is growing. Analyzing cell-free nucleic acids presents a unique opportunity for longitudinal follow-up during treatment of cancer patients. Initiatives have begun to pursue the standardization of methods for cell-free DNA analysis. Understanding the impact of different pre-analytic factors will help accelerate the process and drive large-scale cross-center validation studies to provide robust evidence for clinical utility of circulating tumor DNA and its integration into routine clinical practice.

## Supplemental Data

Supplemental Figure S1 The numbers of circulating tumor DNA (ctDNA) copies, expressed as copies/mL plasma, in different storage and processing conditions (**A**) and the ratio from the sample collected in K_3_EDTA tubes and processed immediately (E.RT.0h) (**B**).

Supplemental Figure S2 Mutant allele fractions of matched samples from six patients, those collected in K_3_EDTA tubes and processed immediately versus those collected in BCT and processed after 1 week of delay.

Supplemental Figure S3 Pairwise comparison between copy number profiles of plasma samples processed with different protocols. EDTA indicates the sample was collected in K_3_EDTA tube and processed immediately; BCT post indicates the sample was collected in BCT and delivered in the post and processed upon arrival; BCT indicates the sample was collected in BCT and stored at room temperature until processing with the posted samples. The copy number data were expressed as the log_2_ ratio of the segmented bin counts. The pairwise comparisons were assessed by Spearman correlation, and the correlation coefficients are indicated on top of each panel.

Supplemental Figure S4 The effects of shipping using cell-free DNA BCT on global copy number alterations from sWGS data (0.175× fold coverage). Shallow whole-genome sequencing data of an ovarian cancer patient's (P227) plasma sample processed with three different protocols: collection in K_3_EDTA tube and processed immediately (**A**); collection in BCT, stored at room temperature, not processed until the posted sample arrived (**B**); collection in BCT, delivered in the post and processed upon arrival (**C**). The data were expressed as the log_2_ ratio of segmented bin counts across the 23 chromosomes. The **red lines** indicate genomic partitions of similar copy number, which reflect the average of the Circular Binary Segmentation and Hidden-Markov Model outputs. Blue indicates a statistically significant gain; orange, a significant loss.

Supplemental Figure S5 Estimating noise from the copy number profiles generated by sWGS data. Median of all absolute pairwise differences (MAPD) between coordinate-sorted copy number values for the different protocols. The data points are colored according to patient ID.

Supplemental Table S3

Data Profile
