# Supplementary figures and images for: Effects of Collection and Processing Procedures on Plasma Circulating Cell-Free DNA from Cancer Patients
Source: J Mol Diagn. 2018 Nov;20(6):883–92. doi: 10.1016/j.jmoldx.2018.07.005 (PMC6197164; doi:10.1016/j.jmoldx.2018.07.005)

A

## DIFFERENT TUBE TYPES AND TEMPERATURES

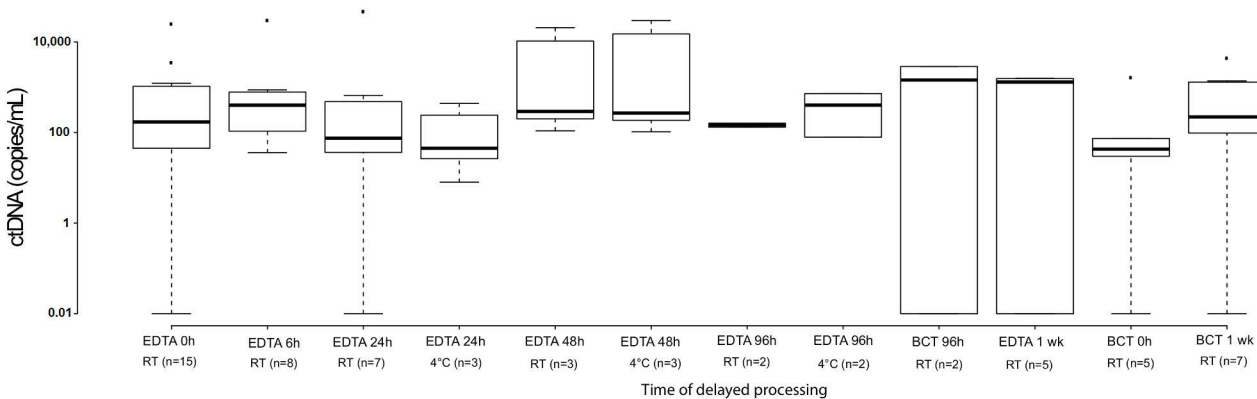

B

## DIFFERENT TUBE TYPES AND TEMPERATURES

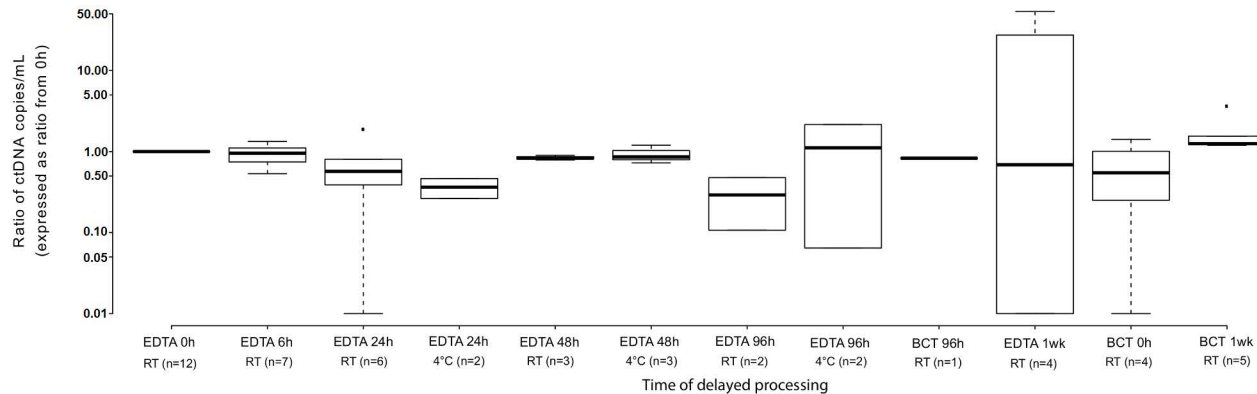

Supplement: Supplemental Figure S1 — The numbers of circulating tumor DNA (ctDNA) copies, expressed as copies/mL plasma, in different storage and processing conditions (A) and the ratio from the sample collected in K3EDTA tubes and processed immediately (E.RT.0h) (B). [file mmc1.pdf]

Mutant allele fraction (%)

8  
6  
4  
2  
0

EDTA  
(0h)

BCT  
(1week)

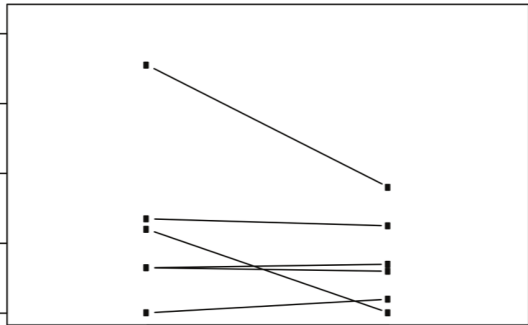

Supplement: Supplemental Figure S2 — Mutant allele fractions of matched samples from six patients, those collected in K3EDTA tubes and processed immediately versus those collected in BCT and processed after 1 week of delay. [file mmc2.pdf]

**BCT (room temperature) vs.  
EDTA**

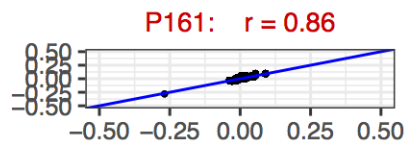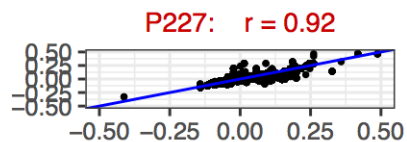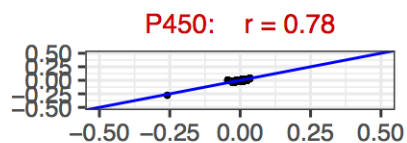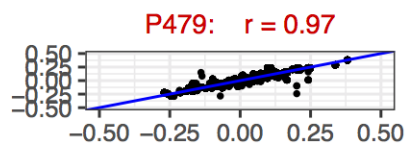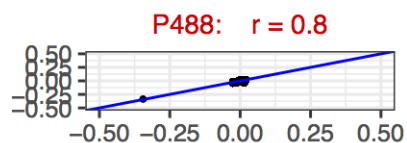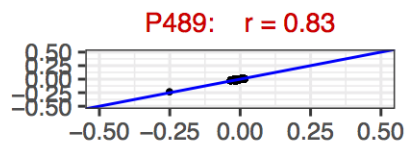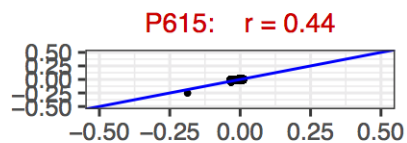

**BCT (Post) vs.  
EDTA**

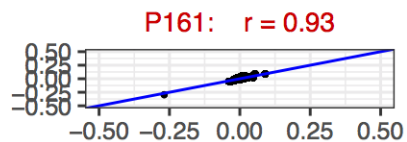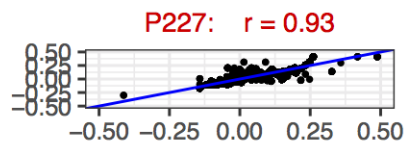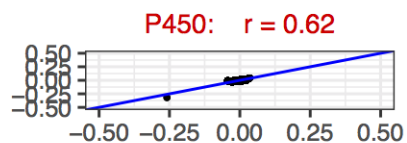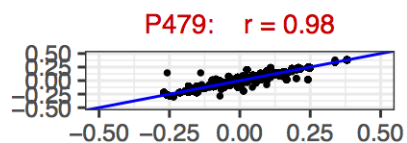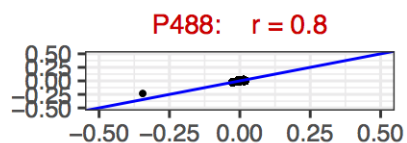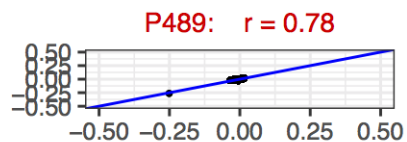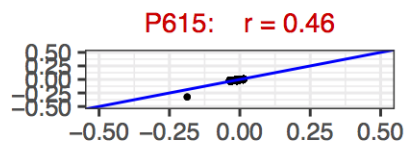

**BCT (room temperature) vs.  
BCT (Post)**

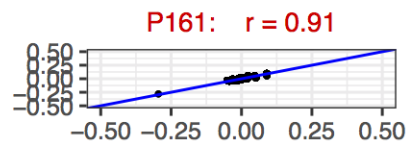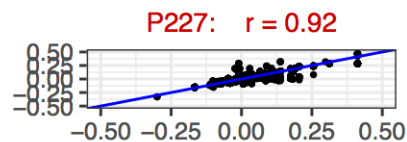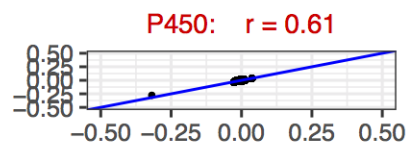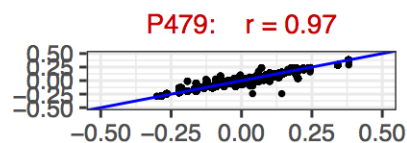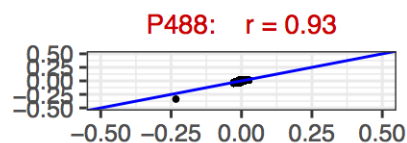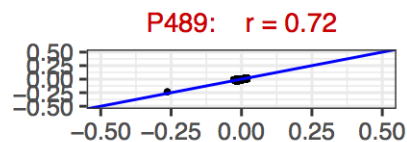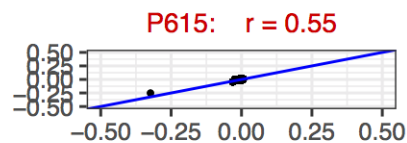

Supplement: Supplemental Figure S3 — Pairwise comparison between copy number profiles of plasma samples processed with different protocols. EDTA indicates the sample was collected in K3EDTA tube and processed immediately; BCT post indicates the sample was collected in BCT and delivered in the post and processed upon arrival; BCT indicates the sample was collected in BCT and stored at room temperature until processing with the posted samples. The copy number data were expressed as the log2 ratio of the segmented bin counts. The pairwise comparisons were assessed by Spearman correlation, and the correlation coefficients are indicated on top of each panel. [file mmc3.pdf]

**A** EDTA, processed immediately

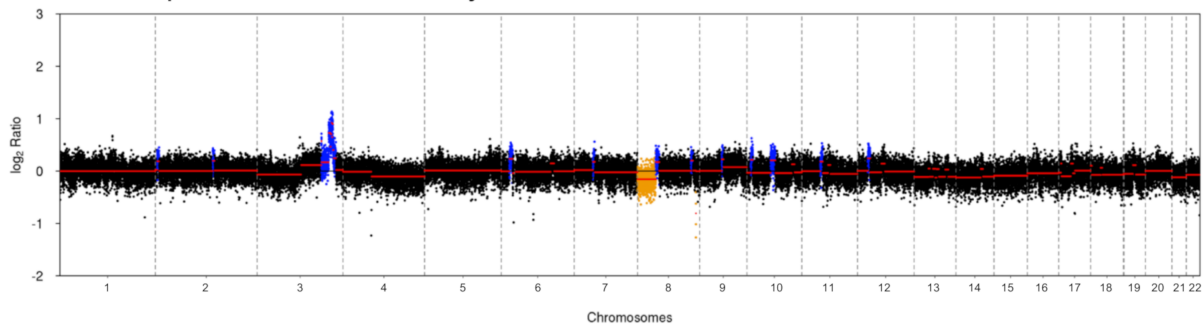

**B** BCT, stored at room temperature until processing

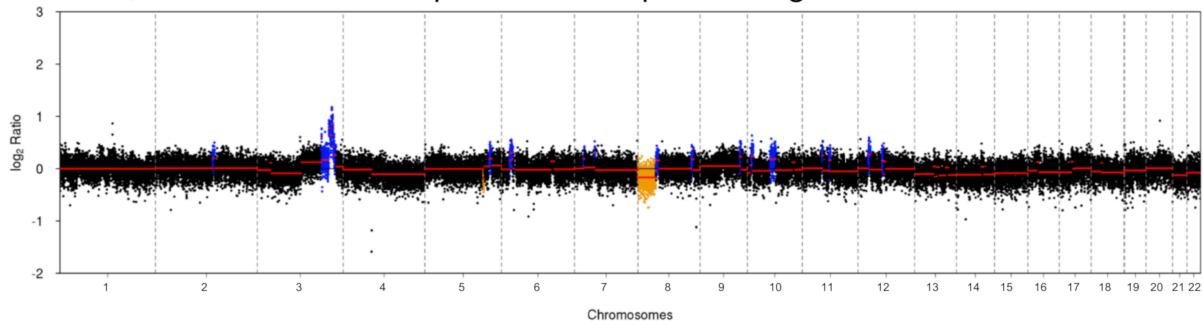

**C** BCT, posted

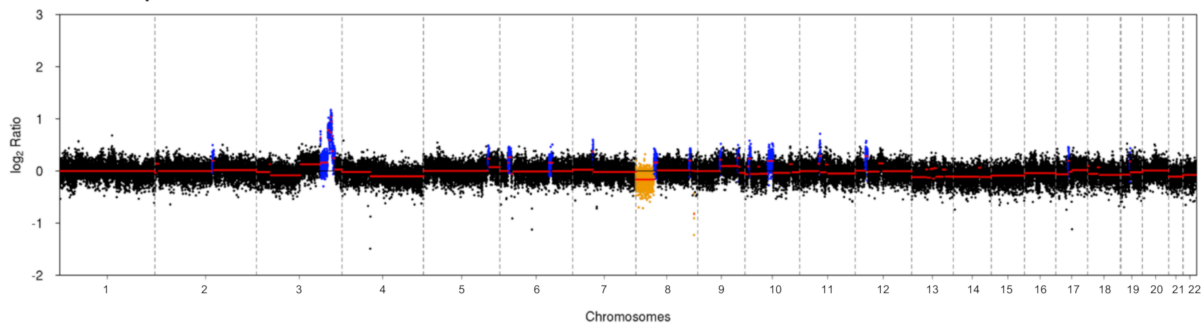

Supplement: Supplemental Figure S4 — The effects of shipping using cell-free DNA BCT on global copy number alterations from sWGS data (0.175× fold coverage). Shallow whole-genome sequencing data of an ovarian cancer patient's (P227) plasma sample processed with three different protocols: collection in K3EDTA tube and processed immediately (A); collection in BCT, stored at room temperature, not processed until the posted sample arrived (B); collection in BCT, delivered in the post and processed upon arrival (C). The data were expressed as the log2 ratio of segmented bin counts across the 23 chromosomes. The red lines indicate genomic partitions of similar copy number, which reflect the average of the Circular Binary Segmentation and Hidden-Markov Model outputs. Blue indicates a statistically significant gain; orange, a significant loss. [file mmc4.pdf]

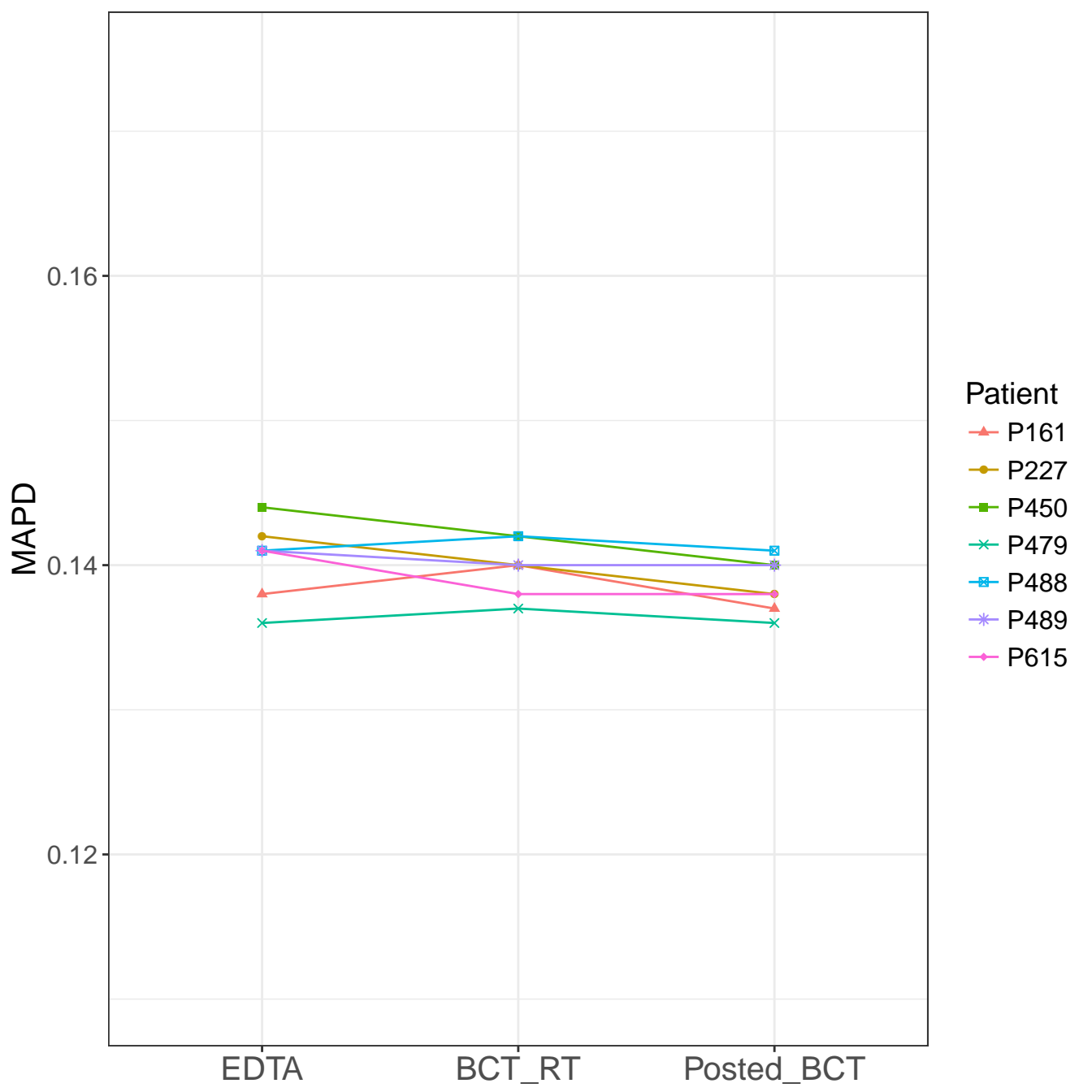

Supplement: Supplemental Figure S5 — Estimating noise from the copy number profiles generated by sWGS data. Median of all absolute pairwise differences (MAPD) between coordinate-sorted copy number values for the different protocols. The data points are colored according to patient ID. [file mmc5.pdf]
