# Supplementary material for: Effects of Collection and Processing Procedures on Plasma Circulating Cell-Free DNA from Cancer Patients
Source: J Mol Diagn. 2018 Nov;20(6):883–92. doi: 10.1016/j.jmoldx.2018.07.005 (PMC6197164; doi:10.1016/j.jmoldx.2018.07.005)
Supplement: Supplemental Table S3 [file mmc8.docx]

|  | **Sample** | **Paired Test** | **Wilcoxon Rank Sum p-value** | **Adjusted**  **p-value** | **Spearman Correlation** |
| --- | --- | --- | --- | --- | --- |
| 1 | P161 | BCT RT vs EDTA | 0.776170358 | 1 | 0.86 |
| 2 | P161 | BCT RT vs Posted BCT | 0.850497698 | 1 | 0.91 |
| 3 | P161 | Posted BCT vs EDTA | 0.790415386 | 1 | 0.93 |
| 4 | P227 | BCT RT vs EDTA | 0.686286315 | 1 | 0.92 |
| 5 | P227 | BCT RT vs Posted BCT | 0.587965029 | 1 | 0.92 |
| 6 | P227 | Posted BCT vs EDTA | 0.779383691 | 1 | 0.93 |
| 7 | P450 | BCT RT vs EDTA | 0.88734619 | 1 | 0.78 |
| 8 | P450 | BCT RT vs Posted BCT | 0.388819676 | 1 | 0.61 |
| 9 | P450 | Posted BCT vs EDTA | 0.283171075 | 1 | 0.62 |
| 13 | P479 | BCT RT vs EDTA | 0.091867971 | 1 | 0.97 |
| 14 | P479 | BCT RT vs Posted BCT | 0.819035363 | 1 | 0.97 |
| 15 | P479 | Posted BCT vs EDTA | 0.101536976 | 1 | 0.98 |
| 16 | P488 | BCT RT vs EDTA | 0.282536243 | 1 | 0.8 |
| 17 | P488 | BCT RT vs Posted BCT | 0.304745255 | 1 | 0.93 |
| 18 | P488 | Posted BCT vs EDTA | 0.823231673 | 1 | 0.8 |
| 19 | P489 | BCT RT vs EDTA | 0.16731823 | 1 | 0.83 |
| 20 | P489 | BCT RT vs Posted BCT | 0.074817827 | 1 | 0.72 |
| 21 | P489 | Posted BCT vs EDTA | 0.844063384 | 1 | 0.78 |
| 22 | P615 | BCT RT vs EDTA | 0.337345169 | 1 | 0.44 |
| 23 | P615 | BCT RT vs Posted BCT | 0.684271396 | 1 | 0.55 |
| 24 | P615 | Posted BCT vs EDTA | 0.749338913 | 1 | 0.46 |

**Supplemental Table S3: Comparison of the segmental copy number profiles among the different collection methods.**

The values for the Wilcoxon Rank Sum Test, adjusted *P*-value, and Spearman correlation for the different comparison of segmental copy number profiles using EDTA, BCT RT, or Posted BCT collection tubes.
